# Screening for coeliac disease in adult patients with type 1 diabetes mellitus: myths, facts and controversy

**DOI:** 10.1186/s13098-016-0166-0

**Published:** 2016-07-29

**Authors:** Sjoerd F. Bakker, Maarten E. Tushuizen, Boudewina M. E. von Blomberg, Hetty J. Bontkes, Chris J. Mulder, Suat Simsek

**Affiliations:** 1 Department of Gastroenterology and Hepatology, VU University Medical Centre, De Boelelaan 1117, 1081 HV Amsterdam, The Netherlands; 2 Department of Pathology, Unit Medical Immunology, VU University Medical Centre, Amsterdam, The Netherlands; 3 Department of Internal Medicine, North West Clinics, Alkmaar, The Netherlands; 4 Department of Internal Medicine, VU University Medical Centre, Amsterdam, The Netherlands

**Keywords:** Coeliac disease, Clinical characteristics, Gluten free diet, Screening, Quality of life, Tissue-transglutaminase antibodies, Complications and type 1 diabetes mellitus

## Abstract

This review aims at summarizing the present knowledge on the clinical consequences of concomitant coeliac disease (CD) in adult patients with type 1 diabetes mellitus (T1DM). The cause of the increased prevalence of CD in T1DM patients is a combination of genetic and environmental factors. Current screening guidelines for CD in adult T1DM patients are not uniform. Based on the current evidence of effects of CD on bone mineral density, diabetic complications, quality of life, morbidity and mortality in patients with T1DM, we advise periodic screening for CD in adult T1DM patients to prevent delay in CD diagnosis and subsequent CD and/or T1DM related complications.

## Background

Coeliac disease (CD) is a permanent intolerance to ingested gluten resulting in immune mediated inflammatory damage to the small intestinal mucosa and a subsequent malabsorption syndrome [1]. Diagnosis of CD requires duodenal biopsy when the patient is on a gluten-containing diet and for the vast majority of adult patients also positive serology [2]. CD is one of the commonest lifelong disorders encountered in Western countries with a prevalence of about 0.6 % in the general population [3] and is, in particular in genetically susceptible individuals, associated with other autoimmune disorders including type 1 diabetes mellitus (T1DM) and autoimmune thyroiditis [4]. T1DM is characterized by T-cell mediated destruction of the insulin-producing β-cells in the pancreas leading to hyperglycaemia and diabetic ketoacidosis [5]. Diabetes is diagnosed based on 1) plasma glucose criteria, either the fasting plasma glucose (FPG) or the 2-h plasma glucose (2-h PG) value after a 75-g oral glucose tolerance test (OGTT) or 2) on a glycated haemoglobin (HbA1c) value of >6.5 % [6]. Long term diabetic complications consist of micro- and macrovascular disease, which account for the major morbidity and mortality associated with T1DM [7]. Up to one-third of patients with T1DM have thyroid antibodies, and half of these patients may progress to clinical autoimmune thyroid disease [8]. The need for annual screening for thyroid disease in T1DM patients has therefore been recommended.

The over all prevalence of CD in T1DM patients is about 6 % [9]. The association between CD and T1DM was first noted over 40 years ago in children [10]. Therefore, screening in paediatric T1DM patients is advocated. However, international paediatric consensus based guidelines differ in the need and frequency of screening for CD [11]. Some recommend an annual screening interval by testing antibodies against tissue transglutaminase 2 (TG2A), others advice to perform these tests in the presence of typical CD symptoms only [11]. However, despite the high prevalence of CD in T1DM patients there is no consensus on screening adult T1DM patients for CD.

In this review it is discussed whether screening for CD should be performed in adult T1DM patients and at which interval. For this purpose, the current literature was screened with respect to the clinical features of patients with both diseases as compared to patients with T1DM alone.

## Association between CD and T1DM

### Genetics

T1DM and CD are auto-immune, inflammatory diseases for which the major genetic contribution arises from the major histocompatibility complex [12]. These so-called HLA-DQ heterodimers enable the presentation of peptides that are derived from otherwise innocuous self- or non-self antigens (proteins from insulin producing beta cells in T1DM, gliadins in CD) and activate pathogenic effector T-cells [13]. Besides the genetic overlap in the major histocompatibility complex, genome wide association studies (GWAS) in these two diseases have revealed a large number of well validated, non-HLA genetic risk loci providing an opportunity to explore the possibility of overlapping susceptibility between them [12].

Thus, genetic overlap exists between CD and T1DM consisting of both HLA and non-HLA genes [14–16]. Both disorders are associated with the major histocompatibility complex (MHC) class 2 antigen DQ encoded by the alleles DQA1*05 with DQB1*02 (DQ2.5) and DQA1*03 with DQB1*03:02 (DQ8) [1, 17].

In patients with CD, individuals who are HLA-DQ 2.5 homozygous have a greater risk of developing CD and the gluten specific T-cell response is more vigorous when gluten peptides are presented by antigen presenting cells homozygous for HLA-DQ 2.5 [18, 19]. In European Caucasian populations, more than 90 % of CD patients carry the HLA-DQ 2.5 heterodimer and the majority of CD patients who do not carry this HLA-DQ 2.5 heterodimer are HLA-DQ8 or HLA-DQ2.2 positive [20].

The main determinant of risk of developing T1DM is HLA-DQ8 and to a lesser extent HLA-DQ 2.5 [21, 22]. In a recent study, we compared the frequency of HLA-DQ haplotypes between 2472 T1DM patients versus 483 T1DM + CD patients [16]. In patients with T1DM, the HLA-DQ 2.5 haplotype showed a significant association and provided the highest risk for developing double autoimmunity (OR = 1.44, p-value = 0.0003, Table 1). As expected, the absence of the haplotypes HLA-DQ 2.5, DQ8 and DQ 2.2 (which is classified as “other” which is present in about 25 % of T1DM patients), showed the strongest protection (OR = 0.66, p = 0.0001, Table 1). Therefore, an HLA-DQ 2.5 negative T1DM patient does not require monitoring for CD.

**Table 1:** Haplotype and genotype HLA association and frequency comparison between double autoimmunity versus type 1 diabetes-only [16]

	T1DM + CD versus T1DM			OR (CI 95 %)	p value
	Frequency controls	Frequency T1DM + CD	Frequency T1DM only		
Haplotype					
DQ 2.5	0.14	0.446	0.318	1.442 (1.189, 1.748)	0.0003
DQ 2.2	0.094	0.046	0.040	1.201 (0.793, 1.821)	0.381
DQ8	0.1	0.346	0.392	0.939 (0.779, 1.131)	0.520
Other	0.663	0.163	0.249	0.660 (0.530, 0.821)	0.0001
Genotype					
DQ 2.5/DQ 2.5	0.020	0.168	0.066	1.20 (1.14, 1.26)	0.0005
DQ 2.5/DQ 2.2	0.032	0.039	0.017	1.16 (1.06, 1.27)	0.242
DQ 2.5/DQ8	0.027	0.350	0.377	0.98 (0.95, 1.01)	0.681
DQ 2.5/other	0.184	0.168	0.112	1.07 (1.03, 1.11)	0.688
DQ 2.2/DQ 2.2	0.012	0.004	0.002	1.18 (0.88, 1.58)	0.169
DQ 2.2/DQ 8	0.022	0.033	0.036	0.98 (0.92, 1.06)	0.908
DQ 2.2/Other	0.111	0.010	0.025	0.91 (0.83, 0.99)	0.326
DQ 8/DQ 8	0.009	0.083	0.078	1.00 (0.96, 1.05)	0.886
DQ 8/Other	0.135	0.143	0.216	0.94 (0.91, 0.97)	0.175
Other/Other	0.449	0.002	0.072	0.84 (0.80, 0.89)	0.028

*CD* coeliac disease, *OR* Odd’s ratio, *T1DM* type 1 diabetes mellitus

In addition to the overlap between T1DM and CD in HLA genes, it was revealed that non-HLA genes overlap as well [12, 16]. *CTLA*-*4* and *IL2RA* loci are more strongly associated with double autoimmunity than with either T1DM or CD alone [16]. The combination of HLA and non-HLA variants might improve risk prediction for potential CD [23].

### Environmental factors

Several environmental factors have been investigated as precipitating factors for the development of T1DM or CD. A popular theory, based on possible molecular mimicry, is the association between autoimmune diseases and viral infections. Prime viral candidates that have been shown to cause precipitation to T1DM are enteroviruses, more specifically Coxsackie viruses [24]. Moreover, rotavirus infection increases the risk for developing T1DM and an association between rotavirus and increased risk for CD has been described as well [25, 26]. Furthermore, an altered composition of bacteria in the gut, altered gut permeability and intestinal inflammation seem to be factors that contribute to the development of T1DM [27]. Exposure to cereals has been described as a risk factor for the development of both T1DM and CD related autoantibodies. However, these studies show conflicting results [28–30].

## Demographic characteristics

### Epidemiology

**Fig. 1:**
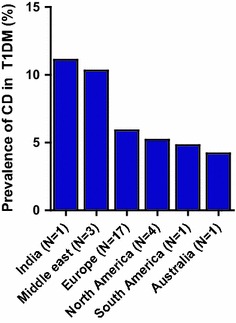
Mean prevalence of screen detected coeliac disease (CD) in children and adults with type 1 diabetes mellitus (T1DM) around the world. Mean prevalence is calculated from studies with at least 100 patients with T1DM [9].* N* indicates the number of screening studies performed on each continent

Many studies have investigated the prevalence of CD in paediatric and adult T1DM patients by different serological screening methods (gliadin, anti endomysium (EMA), anti tissue transglutaminase (TG2A) and anti reticulin antibodies). The prevalence of CD in T1DM patients (children and/or adults) is reported to vary between 0.8 % and 16.4 % with a mean prevalence of 6 % [4, 9, 31]. A large meta-analysis identified 27 studies, which included in total 26 605 individuals with T1DM [9]. Seventeen studies were performed in Europe, 4 in North America, 1 in South America, 1 in Australia, 3 in the Middle East and 1 in India (Fig. 1) [9]. A remarkable high prevalence of CD in T1DM patients is seen in studies performed in Algeria (16.4 %), India (11.1 %) and Saudi Arabia (11.3 %) [32–34]. The relatively high frequency of HLA-DQ 2.5 in the Middle East and India possibly contributes to the high prevalence of CD in T1DM [35]. Furthermore, these countries have a per capita wheat consumption that ranks among the highest in the world [35]. This high prevalence still needs to be confirmed in additional studies. Data from East-Asian and African T1DM cohorts and CD screening are lacking in current literature.

### Clinical presentation

The clinical presentation of CD in T1DM patients resembles that in non-T1DM patients and consists of gastrointestinal complaints (diarrhoea, constipation, vomiting, abdominal distension, anorexia) or extra-intestinal complaints such as growth failure, anaemia, decreased bone mass or osteoporosis, and dental enamel defects [4]. However, CD patients might also be asymptomatic and may have subtle complaints indicative of CD and may only be recognized in retrospect following the benefits of a GFD [36]. Previous studies have reported that 45–60 % of patients with T1DM and CD did not have any complaints of CD indicating a diagnostic challenge [37, 38].

Furthermore, gastrointestinal complaints are common in T1DM patients and a broad differential diagnosis exists for these patients (Table 2) [39, 40]. Furthermore, the fact that a large part of patients presents only with mild symptoms or seem to be asymptomatic provides difficulties for detecting CD [41]. Often, a reduced health is only recognized retrospectively, following the benefits conferred to a GFD [36].

**Table 2:** Differential diagnosis of gastrointestinal complaints in T1DM patients [39, 40, 105, 106]

Causes of gastrointestinal complaints in T1DM patients
Coeliac disease
Diabetic gastropathy
Gastroesophageal reflux disease
Mesenteric ischemia
Irritable bowel syndrome
Hyperglycaemia affects GI motor function and perceptions of the GI tract
Metformin use
Depression
Eating disorders

It has been demonstrated that the risk of CD in T1DM patients is associated with age of onset of T1DM. Children with age of onset of T1DM younger than 4 years are at higher risk to develop CD than those with older age of onset [42]. Regarding clinical practice, we observed two peaks in the age of CD diagnosis in T1DM patients: around 10 and 45 years of age [41]. T1DM diagnosis precedes CD diagnosis in about 90 % of patients and females with T1DM have a higher risk of the additional diagnosis of CD than males [41, 42].

A new syndrome of gluten intolerance, non coeliac gluten sensitivity (NCGS), has been described. NCGS can be diagnosed in those patients with gluten intolerance who do not develop antibodies that are typical neither of CD nor of wheat allergy and who do not suffer from lesions in the duodenal mucosa [43]. Although disease characteristics of NCGS are overlapping with irritable bowel syndrome (IBS), a recent study observed that an associated autoimmune disease was present in 14 % of patients with NCGS, which was mainly autoimmune thyroiditis and sporadically T1DM [44].

### Adherence to a GFD

Nutrition therapy is an important issue in the management of T1DM and the cornerstone of treatment in patients with CD [6, 45].

In T1DM, dietary interventions aim to maintain blood glucose, blood pressure, lipid levels and body mass index in the normal range [46]. A GFD together with an insulin therapy integrated into an individual’s dietary and physical activity pattern imposes practical limitations and leads to restrictions in the lifestyle of a child or adolescent. Therefore, it may not be surprising that non adherence to a GFD in T1DM patients with CD is more common than in CD patients [47, 48]. Another problem that arises is the availability of gluten free food. In 5 different US states it was found to be significantly less available than food containing gluten [49]. The increased cost of GFD products may have an impact on compliance in T1DM patients with CD as well [49]. Therefore, we advise that patients with both conditions are guided by a skilled dietitian.

## Clinical consequences of CD in adult patients with T1DM

So far, studies addressing the consequences of CD in adult T1DM patients differ in methodology, study size and prospective/retrospective design. Therefore, these results are difficult to compare and interpret. An overview of these results is given in Table 3.

**Table 3:** Clinical consequences of coeliac disease (CD) in adult Type 1 Diabetes Mellitus (T1DM) patients as compared to T1DM without CD

Clinical consequence	T1DM + CD	Patients on GFD	References
HbA1c	Hba1c in screen detected CD patients is lower (Kaukinen, Bakker), higher (Leeds)	NA	[50]
		NA	[51]
		NA	[59]
	No difference in HbA1c during follow up	Yes	[51]
		Yes	[54]
	No increased risk for hospital admission due to hypoglycaemia, keto-acidosis or coma	Unknown	[52]
Cholesterol + triglycerides	Lower in screen detected CD patients	NA	[59]
		NA	[54]
Nephropathy	Higher prevalence of nephropathy	Unknown	[65]
		Unknown	[64]
Retinopathy	<10 years of CD results in less retinopathy, more than 10 years leads to more retinopathy	Unknown	[60]
		Yes	[51]
Bone mineral density	Lower BMD at diagnosis	NA	[71]
Quality of life	Decrease, particularly in women, both social functioning and general health perception are affected	Yes	[75]
Depression	Increased risk	Unknown	[77]
Refractory Coeliac disease	?		?
Enteropathy associated T cell lymphoma	?		?
Mortality	A diagnosis of CD for >15 years increases the risk of death in patients with T1D	Unknown	[82]

?, no studies performed; NA, not applicable

### Glycaemic control

In adult patients with T1DM, no significant change of HbA1c levels was found, when comparing before CD diagnosis, at CD diagnosis and after treatment of CD by a GFD [50, 51]. This data is confirmed in a recent population based cohort study which found that having a diagnosis of CD does not influence the risk of hospital admission due to hypoglycaemia, keto-acidosis or coma in T1DM patients [52].

### Lipid profile

Undetected CD in the general population is associated with lower cholesterol levels, which is thought to contribute to a favourable cardiovascular risk profile in untreated CD patients [53]. Accordingly, lower levels of cholesterol and triglycerides were found in newly detected, untreated CD patients with T1DM [54]. The assumed mechanism that may contribute to the lower cholesterol levels in undetected CD patients is malabsorption.

### Microvascular complications

Intensive insulin therapy to normalize blood glucose levels effectively delays the onset and slows the progression of microvascular complications including diabetic retinopathy, nephropathy and neuropathy in T1DM patients [55–57]. Several studies investigated the influence of (newly diagnosed) CD with or without treatment by a GFD on long term diabetic complications and found CD to be either protective [51, 54, 58] or aggravating [59–61]. A recent large nationwide study in Sweden revealed that the duration of CD is important for the eventual effect [60]. They showed that individuals with T1DM and CD were at a lower risk of diabetic retinopathy in the first 5 years after CD diagnosis (adjusted hazard ratio (HR) 0.57 [95 % CI 0.36–0.91]), followed by a neutral risk in years 5 to <10 years (1.03 [0.68–1.57]). With longer follow-up, coexisting CD was a risk factor for diabetic retinopathy (10 to <15 years of follow-up, adjusted HR 2.83 [95 % CI 1.95–4.11]; ≥15 years of follow-up, 3.01 [1.43–6.32]) [60]. They ascribe the protective effect in the first 5 years to lower cholesterol levels and lower body mass index (BMI). However, this study lacks individual-based information on GFD adherence.

In a study of our group we found less diabetic retinopathy in a T1DM population with a mean CD duration of 3 years+ treatment by GFD compared to T1DM patients without CD [51]. Also, a previous study by Pitocco et al. showed more subclinical atherosclerosis in T1DM patients with a mean duration of treated CD of 9.9 years [61]. These studies suggest that a short duration of CD is protective and a longer duration of CD may aggravate diabetic complications [51, 60].

### Renal disease

CD is associated with a higher risk of end-stage renal disease (ESRD) with a Hazard Ratio (HR) for ESRD of 2.87 (95 % CI 2.22 to 3.71, p < 0.001) [62]. The cumulative prevalence of end-stage renal disease in T1DM patients without CD, is 2.2 % at 20 years and 7.7 % at 30 years [63]. Interestingly, in T1DM patients with CD it was found that non-adherence to a GFD was associated with early elevation of albumin excretion in urine, a recognized factor for diabetic nephropathy [64]. Skovbjerg et al. found that there was a higher prevalence of CD in T1DM patients with nephropathy (2.6 %) than in T1DM patients without nephropathy (1 %) [65]. A recent study found a positive association between longstanding CD in T1DM patients and chronic renal disease in T1DM [66]. For chronic renal disease, this excess risk was present after more than 10 years of CD (HR 2.03, 95 % CI 1.08, 3.79) [66]. However, data about GFD adherence was lacking. These studies suggest that concomitant CD in T1DM patients might lead to more nephropathy in case of longstanding CD, in particular in case of poor adherence to a GFD [64]. The underlying mechanisms need, however, to be elucidated.

### Bone mineral density

Decreased bone mineral density (BMD) is observed both in T1DM patients [67] and in CD patients [68]. In the latter group of patients, this is especially related to the intestinal malabsorption of vitamin D, necessary for healthy bone metabolism [68]. Reports have shown that bone mineral density is lower in paediatric T1DM patients with undiagnosed CD than in T1DM patients without CD [69, 70]. As expected, also in adults with both T1DM and active CD, a decreased BMD was found, but whether CD or T1DM was the cause remains unclear [71]. A study by Sategna-Guidetti showed that treatment by a GFD results in an improvement of lumbar spine BMD in adults with CD [72].

In summary, BMD in T1DM + CD patients is generally decreased and follow-up of BMD with possible treatment is warranted. Besides maintaining a GFD, data is scarce whether calcium and vitamin D supplementation in CD patients is mandatory [68]. Lifestyle changes as regular exercise and smoking cessation should be advised, and in the case of osteoporosis, calcium, vitamin D and bisphosphonates should be prescribed [68].

### Quality of life

Both T1DM and CD are chronic illnesses which influence the quality of life (QOL) since the treatments are burdensome and the complications can be debilitating and life threatening. T1DM patients have a diminished QOL which is partly caused by the development of vascular complications [73]. The lower QOL in CD patients is reported especially in the social aspects of life and in those with symptoms, women being mostly affected [74]. In adult T1DM patients with both T1DM and treated CD, we described a compromised QOL particularly in women and both social functioning and general health perception was affected [75]. This is of importance since patients with T1DM are at increased risk of depression [76]. The additional diagnosis of CD further increases the risk of depression, and this should be taken into account in the clinical support of these patients [77].

### Comorbidity and mortality

T1DM is, beside CD, associated with autoimmune thyroid diseases (Hashimoto’s or Graves’ disease) (AIT), autoimmune gastritis, Addison’s disease, and vitiligo [8]. The presence of a third autoimmune disease in T1DM + CD patients is frequently found. A study by Kaspers et al. found a higher incidence of AIT in patients with T1DM and CD (6.3 %) when compared to those with CD alone (2.3 %) [78]. Our clinical practice study in adults revealed that 28 % of T1DM + CD patients were diagnosed with a third autoimmune disease, mainly autoimmune thyroiditis (22 %) [79].

A small group of patients with CD fail to improve clinically and histologically upon elimination of dietary gluten and this complication is referred to as refractory coeliac disease (RCD) [80]. RCD imposes a serious risk of developing enteropathy-associated T-cell lymphoma (EATL). The prevalence of RCD and EATL in the general population is very rare and studies investigating the risk of developing RCD or malignancy in T1DM + CD patients are currently lacking [81].

The question whether CD influences the mortality in T1DM patients was recently investigated in Sweden [82]. These authors described that having a CD diagnosis for more than 15 years was associated with a 2.8-fold increased risk of death in individuals with T1DM [82]. They hypothesized that the excess mortality was caused by persistent low grade inflammation due to CD or poor adherence to a GFD while using insulin therapy.

## Rationale for screening for CD in adult T1DM patients

CD fulfills many of the WHO criteria for screening in patients with T1DM but not all of them [83]. CD is common and well defined, screening tests are simple + safe + accurate, screening seems to be culturally acceptable, treatment is available and clinical detection of CD can be difficult. However, studies are lacking whether screening for CD in T1DM patients is cost effective and it is currently unknown whether screen detected asymptomatic CD patients benefit from starting with a GFD. The latter will be investigated by the CD-DIET study [84] which is designed as a prospective controlled trial in which asymptomatic screen detected CD patients will be treated with or without a GFD. The results of the efficacy and safety of a GFD in patients with T1DM with asymptomatic CD will add significant data to the discussion about screening for CD in T1DM patients [84].

Consequently, there is still no consensus on screening adult patients with T1DM for CD. International guidelines for adult CD and T1DM differ in their recommendations for screening of CD in T1DM patients [2, 6, 85–91] (Table 4). At present, a case-finding approach in adult T1DM patients is most acceptable, ethically and financially [2, 92]. However, a recent study in the United States and Canada underscores the need for an uniform screening program. This study revealed a high variability in testing for CD in T1DM patients together with an inconsistency of management of CD [93]. In addition, we have recently reported that approximately 20 % of patients with T1DM and CD reported to have had CD related complaints for at least 5 years before CD diagnosis was made [79]. The long term consequences of a diagnostic delay are currently unknown. The high prevalence of several complications as reported in Table 3 in T1DM + CD patients, together with improvement of BMD after start of a GFD provides a strong rationale for an uniform screening program together with careful monitoring. Further, a recent randomized study showed that screen-detected and apparently asymptomatic EmA-positive patients at risk for CD benefit from a GFD as measured by extensive clinical, serologic, and histologic parameters [94]. Hypothetically, this data might be extrapolated to asymptomatic CD in T1DM patients. Another argument for screening is the fact that the incidence of T1DM and CD is rising over time [95, 96].

**Table 4:** Clinical recommendations for screening of CD in T1DM patients in adult CD and T1DM guidelines

Guidelines	Years	Recommendation	Reference
CD guidelines			
Gastroenterological Society of Australia	2007	Not reported	[85]
Dutch Society of Gastroenterology	2008	Testing for CD in case of clinical suspicion	[86]
World Gastroenterology Organisation	2013	Not reported	[88]
American College of Gastroenterology	2013	Testing for CD if there are any digestive symptoms, or signs, or laboratory evidence suggestive of CD	[89]
British Society of Gastroenterology	2014	Testing for CD should be performed when CD is suspected	[2]
National Institute for Health and Care Excellence (NICE)	2015	Test for CD at the moment of CD diagnosis and in case of persisting symptoms	[90]
T1DM guidelines			
American Diabetes Association	2014	Screening for CD soon after T1DM diagnosis, thereafter screening should be considered based on signs and symptoms	[6]
National Institute for Health and Care Excellence (NICE)	2015	In case of low BMI or weight loss, screen for CD	[87]
Australian Diabetes Society	2011	Screen for CD at diagnosis and at least in the first five years after diagnosis	[91]

*BMI* body mass index, *CD* coeliac disease, *T1DM* Type 1 diabetes mellitus

**Fig. 2:**
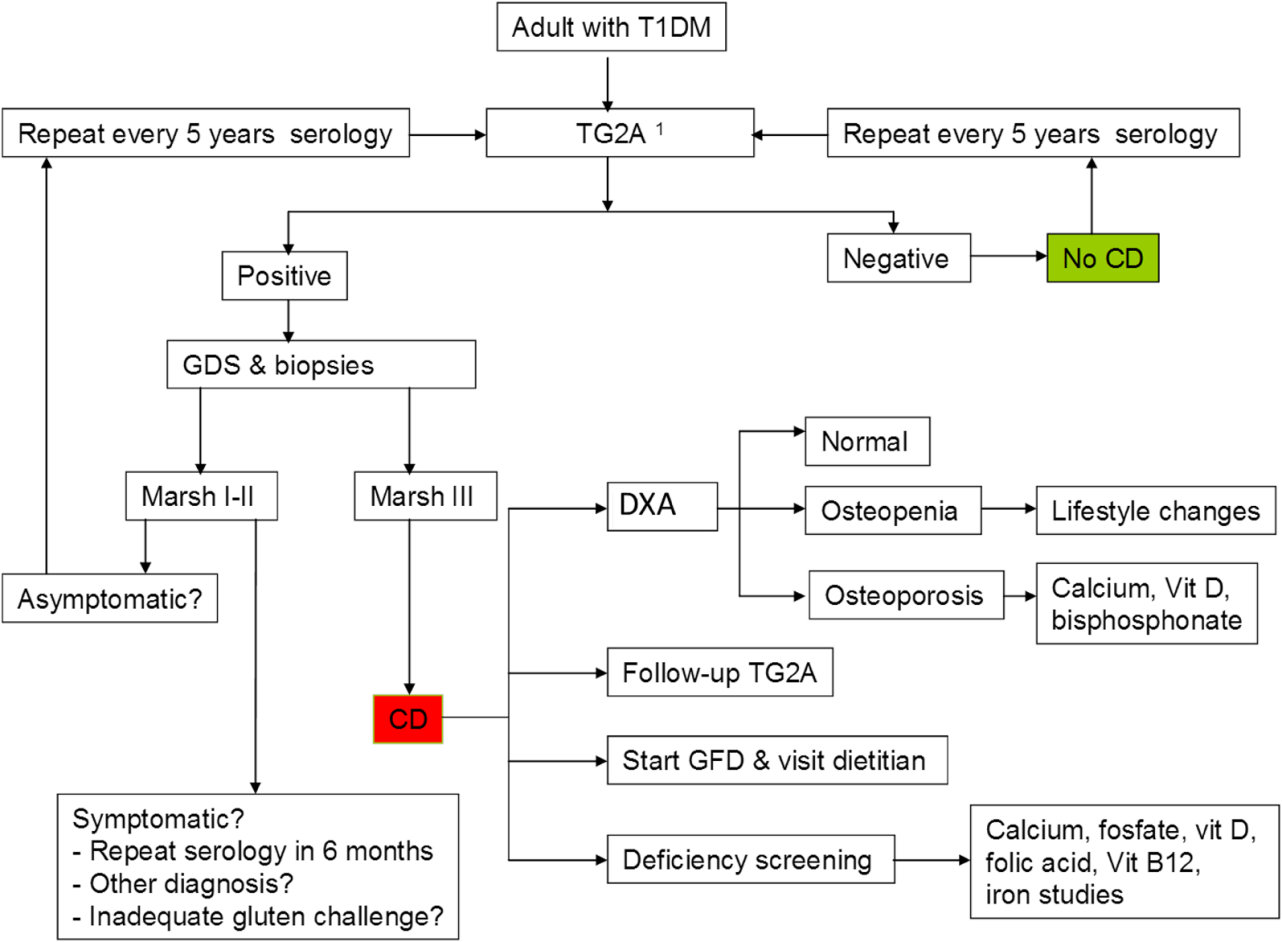
Proposed algorithm for the screening and follow-up of coeliac disease (CD) in asymptomatic patients with type 1 diabetes mellitus (T1DM). *DXA* dual X-ray absorptiometry, *GFD* gluten free diet, *GDS* gastroduodenoscopy, *TG2A* tissue transglutaminase 2 antibodies. *1* IgA TG2A should be evaluated first, in IgA deficient individuals or in patients with high probability of CD IgG TG2A should be performed

We propose the following screening algorithm (Fig. 2) for CD in adult T1DM patients. CD should be diagnosed by serology and duodenal biopsy with the patient on a gluten-containing diet [2]. Serology is by TG2A and if patients are IgA deficient, IgG-TG2A can be used. Villous atrophy (Marsh IIIa- IIIc) is required for diagnosis of CD [2]. Due to the high sensitivity and specificity of TG2A, this test is used for screening in T1DM patients [97]. In case of IgA deficient individuals, or in patients with high probability of CD, IgG TG2A should be tested as 2 % of CD patients are IgA deficient [2]. As T1DM patients might have transient elevations of TG2A, a confirmatory small intestinal biopsy is recommended [98, 99]. In case of a biopsy with Marsh I-II, a serological repetition in 5 year is recommended. Further, another differential diagnosis for intraepithelial lymphocytosis should be considered (e.g. Giardia, olmesartan induced, small intestinal bacterial overgrowth). So far, only retrospective data is available and prospective studies are needed to determine a screening interval for CD in T1DM patients. As proposed by DeMelo et al. [100], we suggest to repeat TG2A testing every 5 years in case of negative serology. A recent systematic review found that most cases of CD are diagnosed within 5 years of T1D diagnosis and they advise screening at T1D diagnosis and within 2 and 5 years thereafter [101]. Only the Australian Diabetes Society recommends screening for CD after 5 years of T1DM diagnosis (Table 4). As studies are lacking investigating the screening frequency in T1DM patients, we advocate continuing screening every 5 years for CD in T1DM patients. In the presence of CD a clinical work-up should be performed to evaluate and possibly treat bone mineral density and vitamin deficiencies (Fig. 2). Based on current data, this screening algorithm is not applicable to all countries as studies about prevalence of CD in T1DM patients are lacking from several countries (Fig. 1).

### HLA-DQ typing

The European Society for Paediatric Gastroenterology, Hepatology and Nutrition (ESPGHAN) guidelines recommend assessing the HLA-DQ2.5/DQ8 genotype in patients with T1DM, as an initial approach for CD screening. A recent study investigated the clinical relevance and cost-effectiveness of human leukocyte antigen (HLA)-genotyping in T1DM patients as a screening tool  [102]. They found that HLA-DQ typing in T1DM patients is neither distinctive nor cost-effective in screening for CD [102]. This might be due to the fact that only 25 % of T1DM patients is HLA-DQ 2.5 or DQ 8 negative [14, 16]. Thus, in our algorithm HLA-DQ typing is excluded.

According to recent guidelines for symptomatic children who have high antibody titres, a duodenal biopsy is not needed anymore for diagnosing CD [103]. Indeed, a recent study showed that none of the T1DM children with high TG2A titres would have needed a biopsy for diagnosis [104]. Whether this is also the case in symptomatic adult T1DM patients with high TG2A titres remains to be established.

## Conclusions

CD fulfills many of the WHO criteria for screening as it is common, simple to diagnose, and treatment is available. Detection of CD in T1DM patients is important as morbidity and mortality is increased in patients with both T1DM and CD. Furthermore, several clinical consequences are present in both disorders as decreased BMD, nephropathy, retinopathy and decreased QOL which need careful follow-up. We propose an algorithm for periodic screening and advise a multidisciplinary approach for these complex patients.

## Abbreviations

AIT: autoimmune thyroid diseases
BMI: body mass index
BMD: bone mineral density
CD: coeliac disease
DXA: dual X-ray absorptiometry
EATL: enteropathy-associated T cell lymphoma
EMA: anti-endomysial antibody
ESPGHAN: European Society for Paediatric Gastroenterology, Hepatology and Nutrition
ESRD: end-stage renal disease
FPG: fasting plasma glucose
GDS: gastroduodenoscopy
GWAS: genome wide association studies
HbA1c: glycated haemoglobin
IBS: irritable bowel syndrome
NCGS: non coeliac gluten sensitivity
OR: odd’s ratio
OGTT: oral glucose tolerance test
QOL: quality of life
RCD: refractory coeliac disease
TG2A: tissue transglutaminase 2
T1DM: type 1 diabetes mellitus
WHO: World Health Organization
